# Reimplantation of Transplanted Kidneys for Salvage of Complex Posttransplant Vascular Lesions

**DOI:** 10.1097/TXD.0000000000001959

**Published:** 2026-08-11

**Authors:** Mahmoud Morsi, Javier González, Vighnesh Venkatasamy, Amay Banker, Ahmad Nasrallah, Armando Salim Munoz-Abraham, Yuri Kim, Gaetano Ciancio

**Affiliations:** 1 Department of Surgery, Miami Transplant Institute, Miami, FL.; 2 Department of Surgery, Miami Transplant Institute, University of Miami Miller School of Medicine, Jackson Memorial Hospital, Miami, FL.; 3 Department of Urology, Gregorio Marañón General University Hospital, Urology Service, Kidney Transplant Unit, Madrid, Spain.; 4 Department of Urology, Miami Transplant Institute, Miami, FL.

Kidney transplantation is widely regarded as the optimal treatment modality for patients with end-stage kidney disease (ESKD).^1^ This field has undergone substantial evolution, driven by advances in surgical techniques, immunosuppressive pharmacotherapy, and perioperative management, all contributing to improvements in graft survival.^2,3^ Despite these gains, kidney transplant recipients remain at risk for a spectrum of complications that can jeopardize graft integrity and function. This includes technical complications, particularly those involving vascular anastomoses.^3^

Among vascular complications, transplant renal artery stenosis (TRAS) is an infrequent but consequential entity, occurring in 1%–5% of recipients.^4,5^ Causes are multifactorial, encompassing technical factors during anastomosis, perioperative trauma, and preexisting vascular abnormalities in the donor.^4^

Management strategies for TRAS must be tailored to lesion characteristics and patient-specific factors. Percutaneous transluminal angioplasty (PTA) is generally considered first-line therapy. However, in cases of early TRAS within 1 mo of transplant, complex vascular anatomy, or extensive arterial injury, surgical alternatives become necessary. Reimplantation of the kidney allograft—a technique first described by Hardy in the 1960s—entails explantation, ex vivo vascular reconstruction, and reimplantation, thereby facilitating precise repair under cold preservation conditions.^6-8^

Herein, we present 2 cases of severe vascular compromise following kidney transplantation that were successfully managed with ex vivo arterial reconstruction and reimplantation. This study was approved by the University of Miami Institutional Review Board and follows the ethical principles of the Helsinki Declaration. All patients gave written informed consent for publication.

## CASE 1

### Presentation and Evaluation

A 33-y-old man with ESKD due to type 1 diabetes underwent simultaneous kidney–pancreas transplantation. Kidney Donor Profile Index was 40%. A left renal allograft was placed in the left iliac fossa with vascular anastomoses to the external iliac vessels; the pancreas was implanted on the right external iliac vessels. The initial ultrasound findings were normal, and he achieved immediate euglycemia and a falling creatinine until postoperative day (POD) 7 (creatinine 2.0 mg/dL). On POD 10, ileus and intra-abdominal fluid prompted emergent laparotomy; both grafts were well perfused.

**FIGURE 1.**
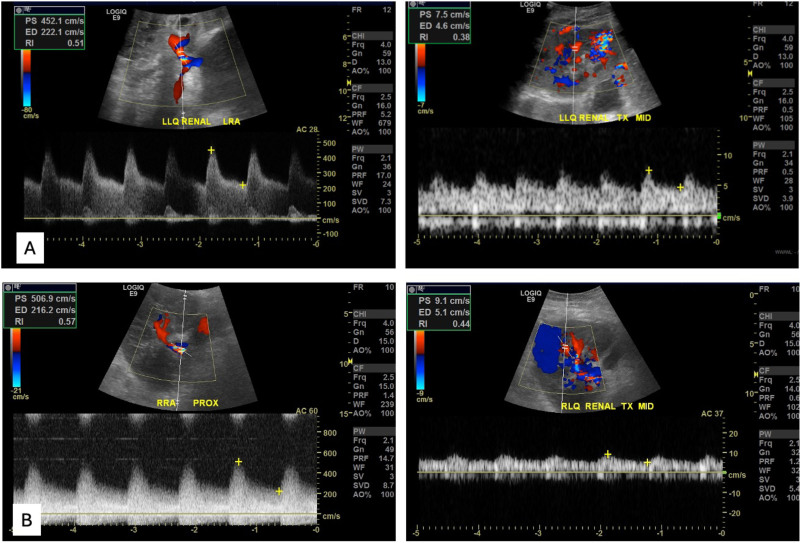
Doppler ultrasound evaluation of transplant renal arteries. A,Case 1 Doppler ultrasound: markedly elevated mid-renal artery velocity and reduced intraparenchymal diastolic flow, consistent with hemodynamically significant stenosis/compression. B, Case 2 Doppler ultrasound: markedly elevated mid-renal artery velocity and reduced intraparenchymal diastolic flow, consistent with hemodynamically significant stenosis/compression.

On POD 13, he developed acute kidney injury (AKI). Doppler ultrasound demonstrated a sizable perirenal collection adjacent to the graft, markedly elevated mid-renal artery peak systolic velocity, and diminished intraparenchymal flow suggestive of hemodynamically significant stenosis and extrinsic compression (Figure 1A). Concerns of a graft compartment syndrome in addition to TRAS prompted immediate surgical intervention.

### Operative Management

At reexploration, we encountered minimal perirenal adhesions, and access to the hilar structures was possible. A fixed narrowing/spasm was identified in the mid-segment of the transplant renal artery, likely secondary to a traction injury during procurement that was not evident at transplant. The artery and vein were clamped at their anastomoses to the external iliac vessels, and the distal ureter was divided. The kidney was explanted and transferred to the back table. Opening the renal artery revealed an injured, narrowed segment ≈1 cm from the ostium. The artery was divided distal to the injury, and the kidney was flushed with ice-cold histidine–tryptophan–ketoglutarate solution.

**FIGURE 2.**
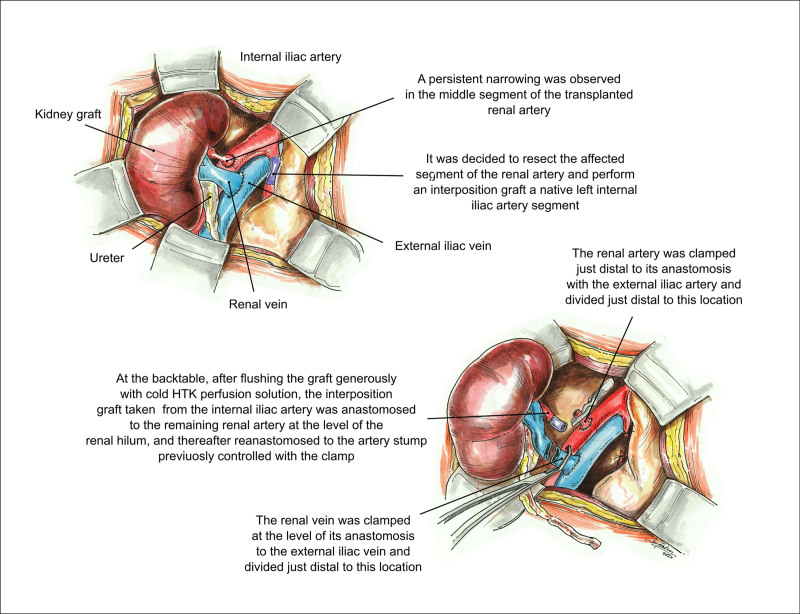
Case 1 intraoperative reconstruction: explantation and back-table resection of the injured renal arterial segment, end-to-end interposition of an autologous left internal iliac artery segment at the hilum, and reimplantation to the external iliac vessels. HTK, histidine–tryptophan–ketoglutarate.

A short autologous left internal iliac artery segment was procured and anastomosed end-to-end to the renal artery at the hilum with running 7-0 polypropylene, thereby restoring length and caliber (Figure 2). The graft was reimplanted: renal vein to the existing venous cuff (6-0 polypropylene), and the reconstructed renal artery to the proximal donor renal arterial stump (7-0 polypropylene). A new extravesical ureteroneocystostomy was performed over a double-J stent. A transplant biopsy and the resected injured arterial segment were sent to pathology. Pathology showed no acute T cell–mediated rejection and moderate acute tubular injury; the excised artery demonstrated serosal/adventitial fibrin.

### Outcome

Reperfusion yielded a firm kidney with excellent turgor and robust pulsatility, followed by immediate urine output. Postoperative imaging revealed good intrarenal arterial waveforms. After transient AKI, creatinine declined to ~1.0 mg/dL, and the patient was discharged with normal graft function (**Figure S1, SDC,** https://links.lww.com/TXD/A872).

## CASE 2

### Presentation and Evaluation

A 66-y-old woman with ESKD from hepatorenal syndrome underwent combined liver-kidney transplantation using grafts from a 23-y-old brain-dead donor (Kidney Donor Profile Index 6%). The right renal allograft was implanted extraperitoneally in the right iliac fossa with a Carrel patch arterial anastomosis to the external iliac artery. The donor kidney had a single artery with early bifurcation and a procurement-related arterial tear primarily repaired with 7-0 polypropylene. Creatinine level fell to 1.1 mg/dL by postoperative week 2.

On POD 21, she developed AKI. Doppler ultrasound on POD 24 showed peak systolic velocity 673 cm/**s** in the proximal renal artery with distal velocities ~19 cm/s and parvus-et-tardus intrarenal waveforms, consistent with high-grade anastomotic TRAS (Figure 1B). A renal angiogram on POD 25 confirmed an irregular, high-grade stenosis distal to the origin with poor distal runoff (**Figure S2, SDC,** https://links.lww.com/TXD/A872). A stent placement was attempted, but we could not navigate across the tight stricture, and the patient was subsequently reexplored.

### Operative Management

**FIGURE 3.**
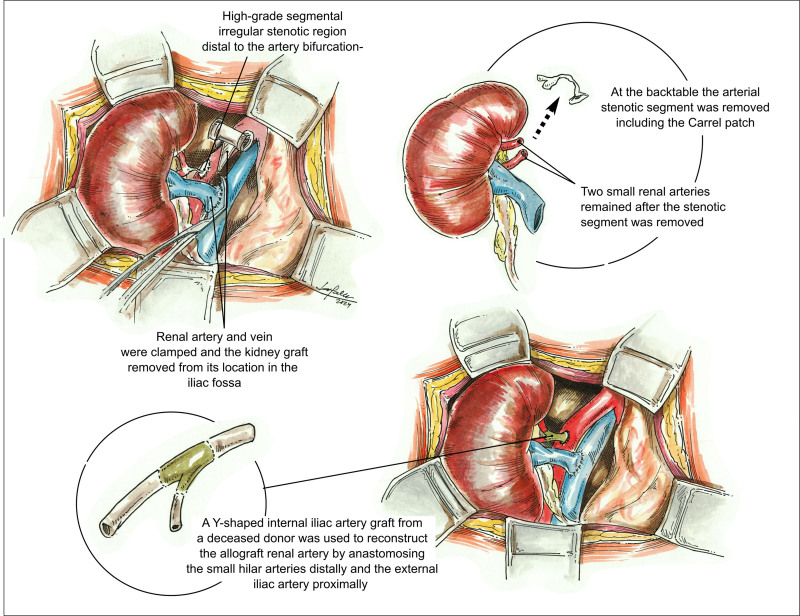
Case 2 intraoperative reconstruction: ex vivo repair of 2 renal arterial branches using branches of a deceased-donor internal iliac artery graft, followed by reimplantation to the external iliac artery.

Given poor intraoperative arterial inflow, the kidney was explanted. On the back table, a short-segment stenosis near the hilum was confirmed at the bifurcation, near the area of the procurement injury. The artery was transected, the kidney flushed with histidine–tryptophan–ketoglutarate, and branches of a deceased-donor internal iliac artery were used to reconstruct 2 renal arterial branches with interrupted 7-0 polypropylene (Figure 3). The reconstructed renal artery was anastomosed to the external iliac artery using the preexisting arteriotomy after excising the Carrel patch; the renal vein was reanastomosed to the existing venotomy site. Reperfusion showed moderate perfusion injury. An extravesical ureteroneocystostomy was recreated, and a double-J stent was placed.

### Outcome

The postoperative course included ~2 wk of AKI followed by steady improvement. Doppler imaging revealed normal arterial flows. Serum creatinine normalized by 6 mo and remained stable at 1 y (**Figure S3, SDC,** https://links.lww.com/TXD/A872).

## DISCUSSION

Vascular complications, particularly TRAS, remain a significant source of morbidity in kidney transplantation despite the steady refinement of surgical techniques and postoperative care.^9^ TRAS can precipitate uncontrolled hypertension, progressive graft dysfunction, and eventual graft loss. Early recognition is paramount; duplex ultrasonography, incorporating velocity criteria and waveform analysis, offers a sensitive, noninvasive screening modality that facilitates timely diagnosis and intervention.^9^

For many patients, PTA remains the preferred initial therapy given its minimally invasive profile.^6,10^ However, lesions located at the arterial hilum, mid-segment fixed stenoses, or those associated with intimal dissection, arterial rupture, or multifocal disease may preclude safe and durable endovascular intervention. In such complex scenarios, surgical revascularization offers a definitive alternative. Ex vivo arterial reconstruction with reimplantation allows meticulous assessment and repair of the affected vessel under cold ischemic conditions, enabling precise excision of the entire diseased segment and interposition of suitable arterial conduits.

Another key factor in determining the optimal management approach is the time-dependent feasibility of surgical reconstruction. Early reexploration—generally within the first 2–3 wk after transplantation—tends to be technically more favorable, as adhesions and perivascular fibrosis are still limited, as demonstrated in our cases. Beyond this period, the development of dense retroperitoneal adhesions and vascular encasement substantially increases operative complexity and risk. Delayed explantation and reimplantation may remain viable in carefully selected patients when performed by experienced transplant surgeons. Timing considerations similarly guide endovascular management. PTA or stent placement is typically deferred until at least 2–3 wk after transplantation to allow stabilization of the arterial anastomosis and to minimize the risk of bleeding or anastomotic disruption. However, in the setting of severe ischemia or impending graft loss, earlier intervention may be warranted, provided it is performed by an experienced interventional radiology team. Our 2 cases illustrate the feasibility and efficacy of this protocol in salvaging at-risk renal allografts in the setting of advanced TRAS or arterial injury.

Procedural success in these cases can be attributed to several factors: (1) prompt recognition of graft hypoperfusion, (2) early surgical intervention before irreversible parenchymal injury, (3) precise microsurgical technique in vessel reconstruction, and (4) concurrent revision of the ureteroneocystostomy to ensure optimal urinary drainage. Importantly, both patients demonstrated complete recovery of graft function and remained free of recurrent stenosis during follow-up, consistent with previous series reporting durable outcomes after kidney reimplantation for complex renovascular disease.^8^

Nevertheless, this approach requires advanced surgical expertise, entails prolonged operative and ischemia times, and carries the inherent risks of a major reoperation. Furthermore, the need for a suitable arterial conduit can be a limiting factor in urgent settings. Therefore, patient selection must be judicious, balancing the operative risk against the likelihood of meaningful graft salvage and long-term benefit.

In summary, although PTA remains the mainstay for most TRAS lesions, ex vivo arterial reconstruction with reimplantation should be considered for early vascular injuries and for anatomically complex or refractory cases. When performed in appropriately selected patients, this approach can restore graft perfusion, preserve long-term function, and avoid the need for retransplantation.

## CONCLUSIONS

When TRAS is anatomically complex or associated with segmental injury, ex vivo vascular reconstruction with reimplantation offers a definitive, kidney-sparing solution. In these 2 cases, timely diagnosis, targeted arterial repair with appropriately sized iliac conduits, and careful reimplantation restored perfusion and preserved long-term allograft function. Broader multicenter data are warranted to refine selection criteria and quantify comparative outcomes versus contemporary endovascular strategies. Supplemental digital content (SDC) is available for this article. Direct URL citations appear in the printed text, and links to the digital files are provided in the HTML text of this article on the journal’s Web site (www.transplantationdirect.com).

## Supplementary Material


