# Supplementary material for: Reimplantation of Transplanted Kidneys for Salvage of Complex Posttransplant Vascular Lesions
Source: Transplant Direct. 2026 Aug 11;12(9):e1959. doi: 10.1097/TXD.0000000000001959 (PMC13466104; doi:10.1097/TXD.0000000000001959)
Supplement: Supplementary file 1 [file txd-12-e1959-s001.pdf]

Supplemental Figures

Supplemental Figure 1

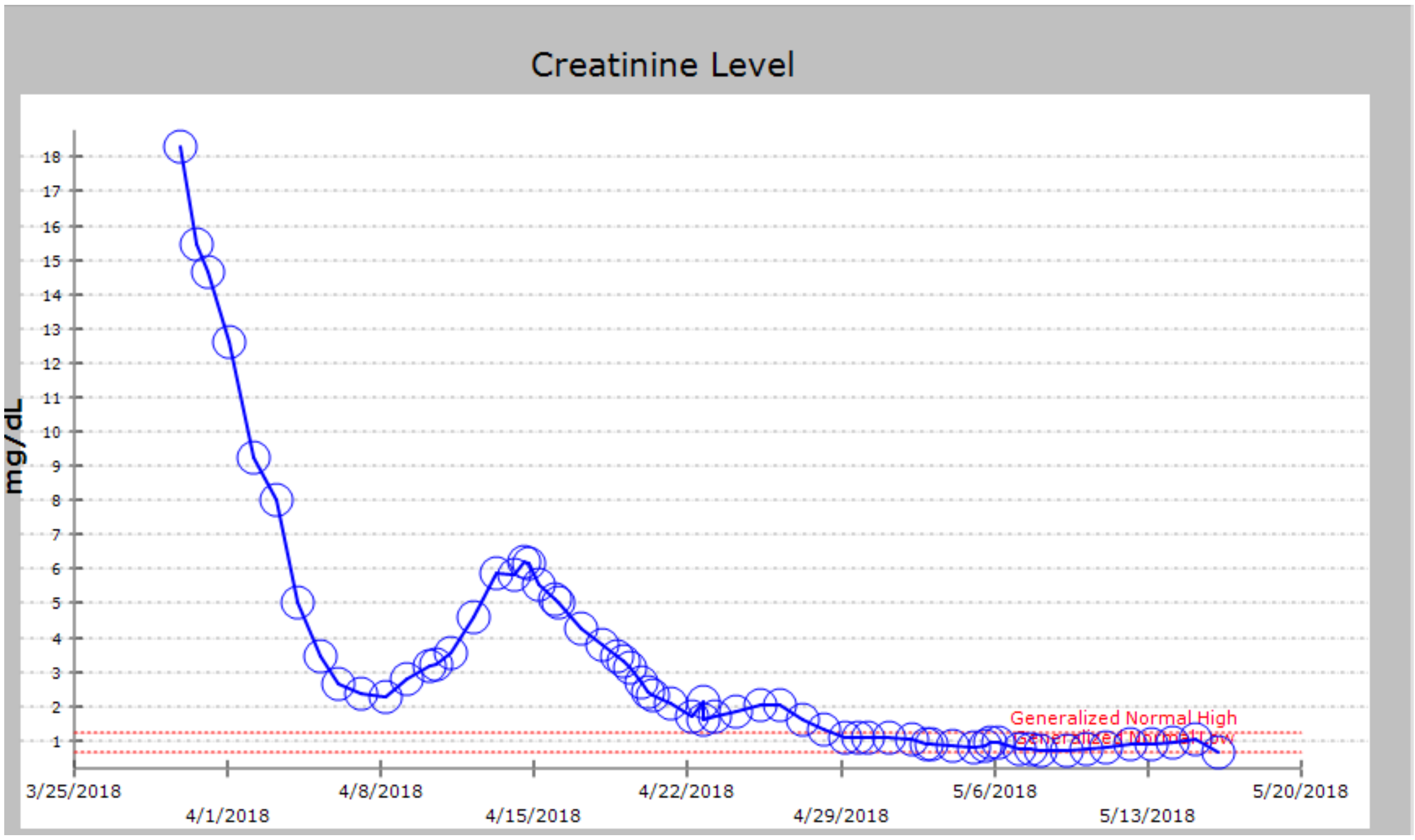

Case 1 serum creatinine trend: early post-transplant improvement, abrupt rise with AKI, and subsequent decline after arterial reconstruction.

Supplemental Figure 2

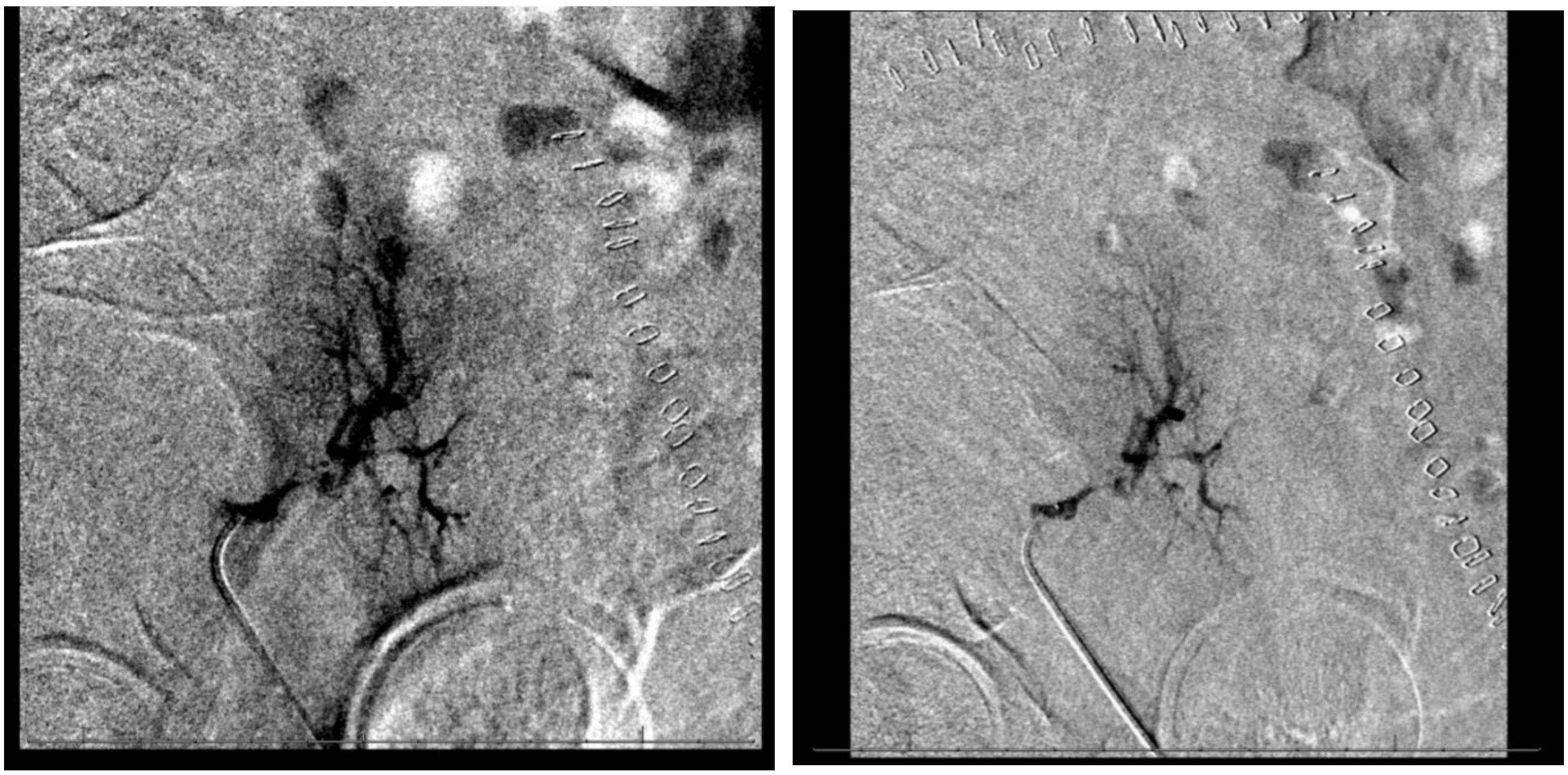

Case 2 renal angiography: high-grade, irregular anastomotic/proximal renal artery stenosis distal to the origin with poor distal runoff.

Supplemental Figure 3

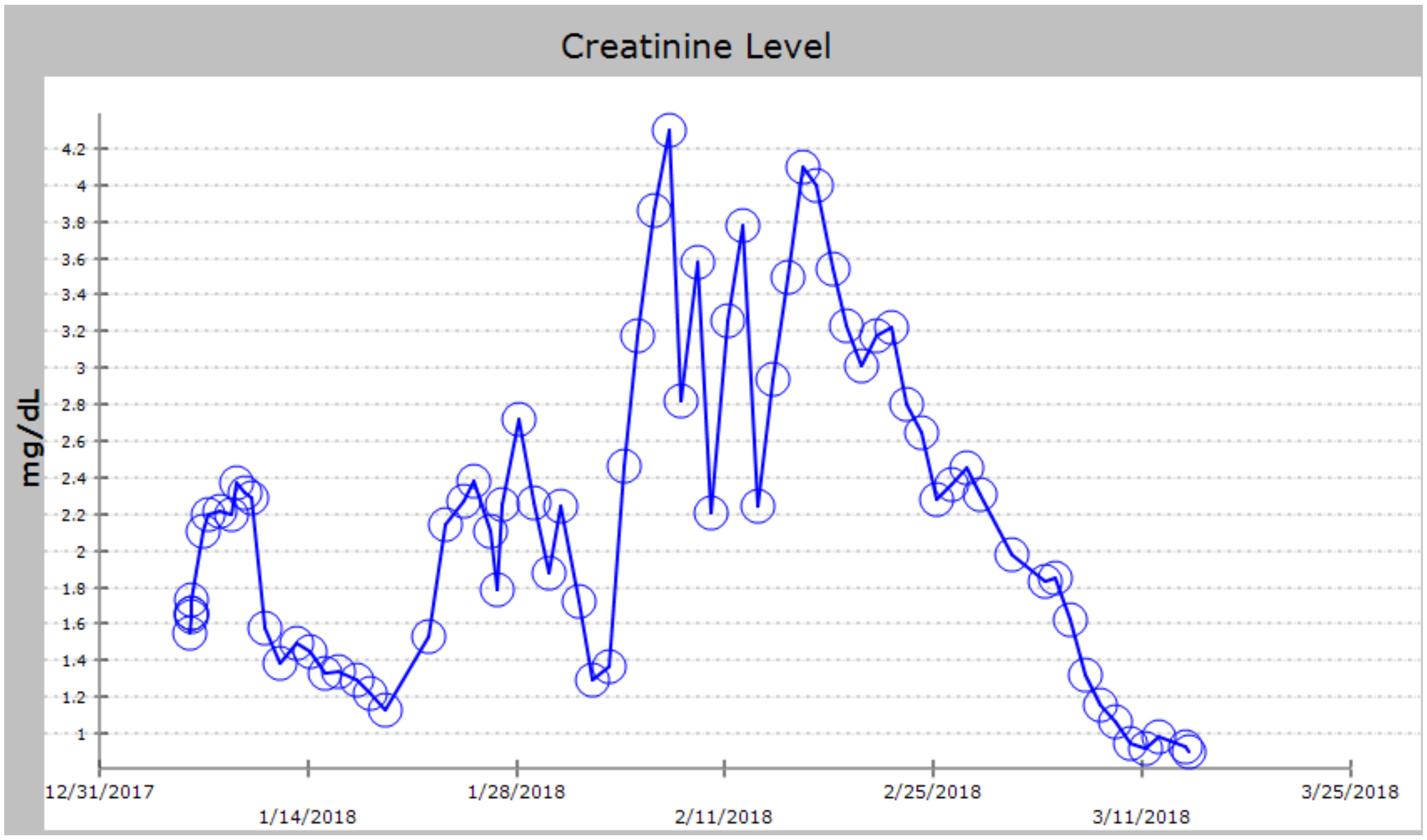

Case 2 serum creatinine trend: AKI around POD 21–35 with recovery; normalization by 6 months and stability at 1 year.
